# Endothelial microparticles delivering microRNA-155 into T lymphocytes are involved in the initiation of acute graft-versus-host disease following allogeneic hematopoietic stem cell transplantation

**DOI:** 10.18632/oncotarget.15579

**Published:** 2017-02-21

**Authors:** Ran Zhang, Xiaoxiao Wang, Mei Hong, Ting Luo, Miaomiao Zhao, Haorui Shen, Jun Fang, Xiaojie Li, Sibin Zang, Ping Chen, Dimin Nie, Peng Zheng, Qiuling Wu, Linghui Xia

**Affiliations:** ^1^ Institute of Hematology, Union Hospital, Tongji Medical College, Huazhong University of Science and Technology, Wuhan, China

**Keywords:** acute graft-versus-host disease, endothelial microparticles, microRNA-155, T lymphocytes, differentiation

## Abstract

Endothelial microparticles (EMPs) upregulation has been observed in acute graft-versus-host disease (aGVHD) after allogeneic hematopoietic stem cell transplantation (allo-HSCT). However, the role of EMPs remains unclear. We found that EMPs derived from TNF-α-stimulated human umbilical vein endothelial cells (EA.hy926) concentrated more microRNA-155 (miR-155) compared with maternal cells. The miR-155 levels in MPs from peripheral blood of aGVHD patients and mice were remarkably elevated and significantly higher than the levels in plasma. Moreover, the rising peak of miR-155 in MPs occurred significantly prior to the peak in T lymphocytes. Additionally, we observed fluorescently-labeled miR-155 in EMPs actively transported into recipient T lymphocytes. Inhibition of miR-155 in EMPs by antagomir-155 did not influence the proliferation and apoptosis of T lymphocytes, but induced defective differentiation toward Th1, Th9 and Th17 cells and skewed differentiation toward Th2 and Treg cells. Furthermore, intravenous injection of miR-155-deficient-EMPs into aGVHD mice significantly attenuated the exacerbation of aGVHD manifestations and abnormal T lymphocytes differentiation induced by high concentration EMPs. Taken together, these data provide a mechanistic framework in which miR-155 delivered by EMPs is involved in aGVHD pathogenesis by activating specific T lymphocytes functions. The results may provide new therapeutic approaches for aGVHD while preserving graft-versus-leukemia (GVL) effect.

## INTRODUCTION

Acute graft-versus-host disease (aGVHD) is still a major contributor to morbidity and mortality following allogeneic hematopoietic stem cell transplantation (allo-HSCT) [1, 2]. However, the detailed pathomechanism underlying the initiation of aGVHD remains poorly understood. Recently, some evidences postulated that endothelial damage played an important role in the initiation of aGVHD [3, 4]. Number of circulating endothelial cells increases during the early phases of conditioning regimens, suggesting that chemotherapeutic drugs induce endothelial damage to release endogenous “danger signals” [5]. Wagner et al. reported that endothelial cells were part of the recipient's antigen-presenting cell compartment and expressed major histocompatibility complex (MHC)-I and II, costimulatory molecules and cytokines that were indispensable for the activation of T lymphocytes [6]. As we know, the activation, cytokine production and expansion of pathogenic alloantigen-specific donor T lymphocytes are hallmarks of aGVHD [1, 7]. However, the mechanism by which endothelial cells substantially activate and modulate T lymphocytes is unknown.

Endothelial microparticles (EMPs) are circulating submicron-sized membranous vesicles released by peripheral blood endothelial cells. Previous work demonstrated that significantly higher levels of EMPs were detected during the aGVHD attack [8–10]. However, the pivotal mechanism underlying the involvement of EMPs in aGVHD has not been definitely clarified. MPs contain cytoplasmic contents such as transcription factors, RNA and microRNAs. If transferred efficiently, these molecules may ultimately influence the phenotype and function of recipient cells [11–13]. Therefore, EMPs may be not only a surrogate marker for endothelial health but also an important transcellular delivery system for the exchange of biological signals necessary for cell-to-cell communication, which may play a crucial role in aGVHD pathological process.

microRNAs are a class of endogenous, highly conserved, noncoding RNAs that negatively regulate gene expression by translational repression or induction of messenger RNA degradation [14]. Recent studies suggested that microRNAs played an integral role in disrupted communication between endothelial cells and immune cells because microRNAs were exported from endothelial cells in a tightly regulated, stimulus-dependent manner [15, 16]. microRNA-155 (miR-155) is a typical multifunctional microRNA that plays a critical role in inflammation regulation and immune response [17, 18]. Recently, miR-155 was shown to be up-regulated in effector T lymphocytes from aGVHD mice and miR-155 in lymphocytes may be essential for lethal mice aGVHD [19]. Intriguingly, miR-155 promotes the proliferation of regulatory T lymphocytes [20]. However, whether and how miR-155 encapsulated within EMPs ultimately influences and activates T lymphocytes during the initiation and development of aGVHD has not been elucidated.

Therefore, in the present study we investigated for the first time the interaction between EMPs and T lymphocytes in the setting of allo-HSCT, which may be a key event in early aGVHD pathogenesis, and identified a potential role for miR-155 delivered by EMPs in the initiation and progression of aGVHD. Our work suggests that miR-155 in EMPs may represent a novel and attractive target for the prevention and treatment of aGVHD following allo-HSCT.

## RESULTS

### miR-155 expressions in MPs, plasma and T lymphocytes from aGVHD and non-aGVHD patients

**Figure 1:**
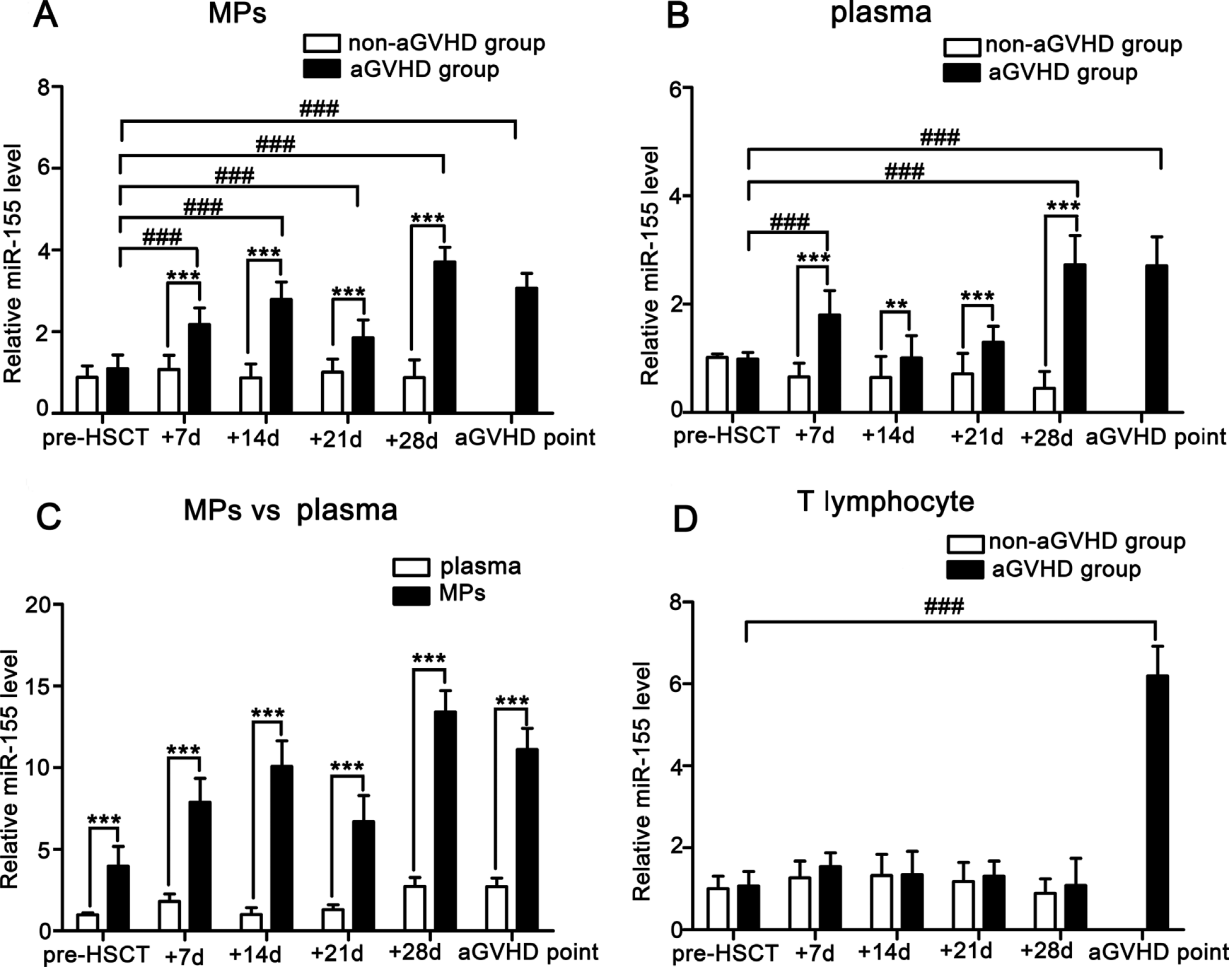
Comparison of miR-155 expressions in MPs, plasma and T lymphocytes from peripheral blood of aGVHD (*n* = 28) and non-aGVHD (*n* = 30) patients Data represent the mean ± SD. (**A**) miR-155 expressions in MPs of aGVHD and non-aGVHD patients. ^**^*P < 0.001, comparison between aGVHD and non-aGVHD at +7d, +14d, +21d, +28d. ^###^*P <* 0.001, miR-155 levels in MPs of aGVHD patients at +7d, +14d, +21d, +28d and aGVHD point were compared with pre-HSCT level. (**B**) miR-155 expressions in plasma of aGVHD and non-aGVHD patients. ^*^P < 0.01, comparison between aGVHD and non-aGVHD at +14d. ^**^*P < 0.001, comparison between aGVHD and non-aGVHD at +7d, +21d, +28d. ^###^*P <* 0.001, miR-155 levels in plasma at +7d, +28d and aGVHD point were compared with pre-HSCT level. (**C**) miR-155 expressions in MPs and plasma from aGVHD patients. ^**^*P < 0.001, miR-155 expressions in MPs pre-HSCT, at +7d, +14d, +21d, +28d and aGVHD point were compared with plasma. (**D**) miR-155 expressions in T lymphocytes of aGVHD and non-aGVHD patients. ^###^*P <* 0.001, miR-155 level in T lymphocytes at aGVHD point was compared with pre-HSCT level.

We firstly compare miR-155 levels in MPs, plasma and T lymphocytes isolated from peripheral blood of aGVHD and non-aGVHD patients at indicated time points. As shown in Figure 1A–1B, the miR-155 levels in MPs and plasma of aGVHD patients were remarkably higher than those in non-aGVHD patients. To investigate whether the blood miR-155 levels were predominantly associated with MPs, miR-155 expressions in plasma and MPs were compared in aGVHD patients. We noted significantly increased expression of miR-155 in MPs compared with plasma (pre-HSCT: 4.023-fold; +7d: 4.366-fold; +14d: 10.014-fold; +21d: 5.126-fold; +28d: 4.913-fold; aGVHD point: 4.103-fold; *P <* 0.001; Figure 1C). Additionally, the miR-155 level in MPs was elevated from +7d and specifically peaked at +28d post-transplant, which was prior to the elevation in T lymphocytes (*P <* 0.001; Figure 1D).

### TNF-α stimulates endothelial cells to release EMPs encapsulating miR-155

**Figure 2:**
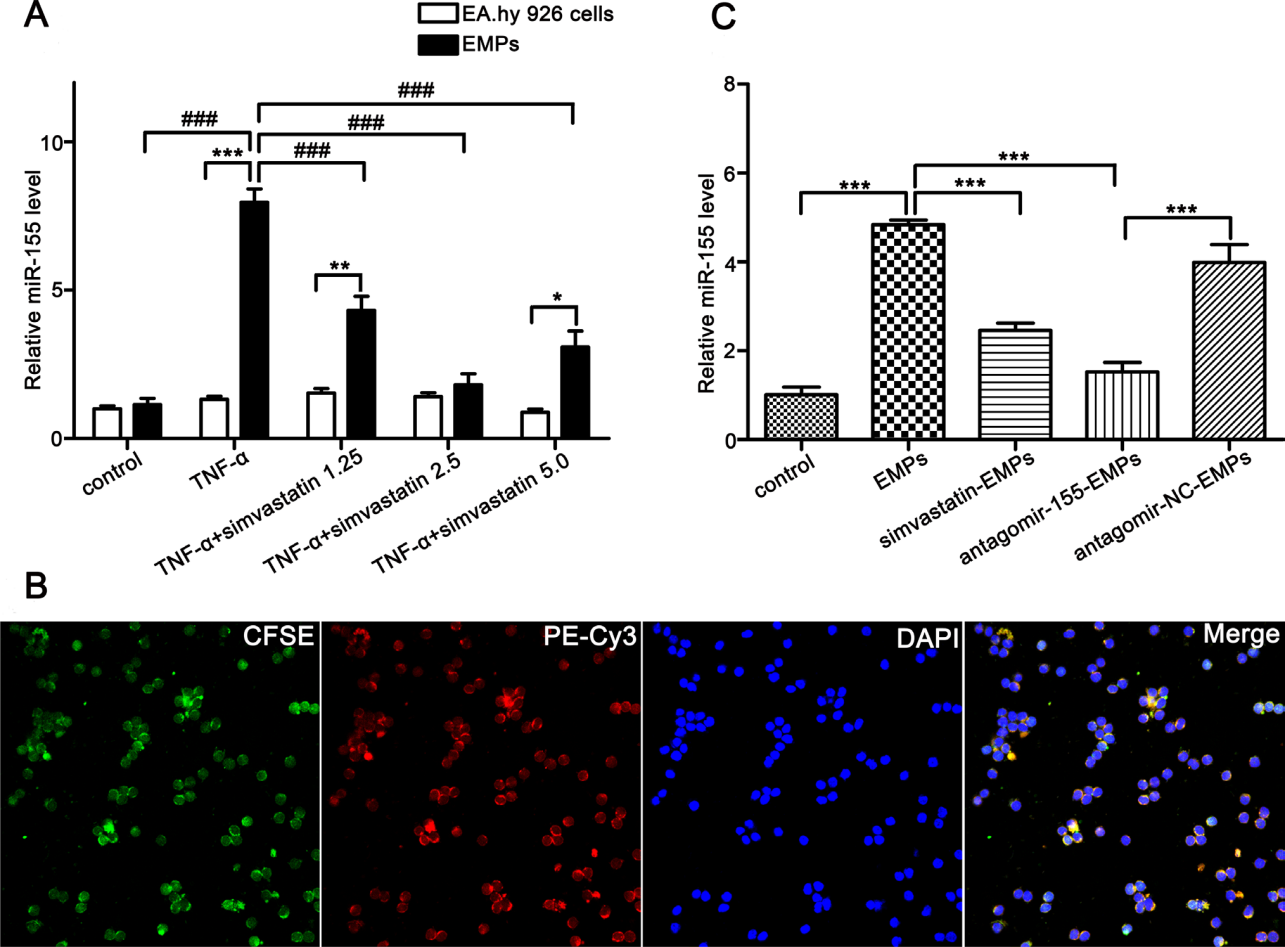
EMPs bind to and merge with T lymphocytes, transfer their miR-155 to T lymphocytes (**A**) miR-155 levels in EMPs (from EA.hy926, TNF-α-stimulated EA.hy926, TNF-α-stimulated EA.hy926 protected by simvastatin of 1.25 μmol/L, 2.5 μmol/L and 5 μmol/L) and maternal cells. *P < 0.05, miR-155 levels in EMPs from TNF-α-stimulated EA.hy926 protected by 5 μmol/L simvastatin and maternal cells. ^*^P < 0.01, miR-155 levels in EMPs from TNF-α-stimulated EA.hy926 protected by 1.25 μmol/L simvastatin and maternal cells. ^**^*P < 0.001, miR-155 levels in EMPs from TNF-α-stimulated EA.hy926 and maternal cells. ^###^*P* < 0.001, Levels of miR-155 in different EMPs were compared. Average values ± SD from three independent experiments were plotted. (**B**) Representative confocal microscopy images of the fusion of T lymphocytes with EMPs (original magnification ×600). T lymphocytes were cultured with CFSE-labeled EMPs from EA.hy926 transfected with miR-155 mimic stained with PE-Cy3 (double fluorescently-labeled EMPs). The PE-Cy3-miR-155 signals were detected in the cytoplasm of T lymphocytes (red), and green signals indicate CFSE-labeled EMPs. PE-Cy3-miR-155 signals are colocalized with CFSE in T lymphocytes (yellow). Nuclear counterstaining was performed using DAPI (blue). (**C**) miR-155 levels in T lymphocytes were determined after incubated with 10 μg/mL EMPs for 3 days. ^**^*P < 0.001. Average values ± SD from three independent experiments were plotted.

The human umbilical vein endothelial cell line is a widely accepted and the most common endothelial cell in multiple vascular and autoimmune diseases studies [21, 22]. TNF-α, which participates in the initiating events that culminate in aGVHD as well as amplifies the disease process once established, is an important mediator and regulator of aGVHD and significantly increases before the appearance of aGVHD in patients undergoing allo-HSCT [23]. Therefore, TNF-α induced HUVECs injury was thought to be able to simulate the course of aGVHD *in vitro* and also adopted by other researchers as an *in vitro* model to study aGVHD [10]. We used TNF-α-stimulated EA.hy926 cells to imitate aGVHD *in vitro*. We previously demonstrated that TNF-α (100 ng/mL, 24 hours) induced EA.hy926 to produce the highest level of EMPs. Herein, TNF-α (100ng/mL) was used to stimulate EA.hy926 to shed EMPs, and miR-155 expressions in EMPs and maternal cells were compared. The EMPs derived from TNF-α-stimulated EA.hy926 was significantly elevated and 2.5 μmol/L simvastatin (an endothelial protector) could reduce the production of EMPs (Supplementary Figure 1). The miR-155 levels in EMPs from TNF-α-stimulated EA.hy926 were significantly higher than those in maternal cells and non-stimulated EMPs (Figure 2A). We measured the level of EMPs after inhibiting miR-155 on EA.hy926 cells using the Bradford method [24] and found that the total content of the secreted EMPs were not altered (antagomir-155: 1.667 ± 0.176 μg/μl vs antagomir-NC: 1.733 ± 0.120 μg/μl, *P* = 0.770). In addition, we have assessed the level of miR-155 in EMPs derived from TNF-α stimulated EA.hy926 cells previously transfected with antagomir-155. We found that the miR-155 was significantly reduced after antagomir-155 treatment to EA.hy926 cells in comparison with antagomir-NC treatment as shown in Supplementary Figure 2. This result reveals that TNF-α can stimulate EA.hy926 cells to produce EMPs encapsulating a large quantity of miR-155.

### EMPs form conjugates with T lymphocytes and transfer miR-155 to T lymphocytes

Given that a number of surface molecules on EMPs have corresponding ligands/receptors on T lymphocytes and to testify the potential of EMPs to act as cell-to-cell communicators, we assessed the ability of EMPs to be taken up and transfer their miR-155 to T lymphocytes. We firstly transferred PE-Cy3 fluorescently-labeled miR-155 mimic into EA.hy926 cells previously stained with CFSE, then collected EA.hy926-derived EMPs and co-cultured with T lymphocytes. Surprisingly, we observed that the miR-155 carried by EMPs entered the cytoplasm of T lymphocytes by confocal laser-scanning microscopy (Figure 2B).

To determine whether EMPs uptake correlated with their ability to deliver miR-155, we knocked down miR-155 in EMPs by transfecting EA.hy926 cells with antagomir-155. We assessed miR-155 levels in recipient T lymphocytes after incubating with miR-155-deficient-EMPs. The expression of miR-155 in T lymphocytes treated with miR-155-deficient-EMPs was remarkably lower than in antagomir-NC-transferred group (*P <* 0.001; Figure 2C).

### miR-155 delivered by EMPs did not modulate the proliferation and apoptosis of T lymphocytes

Because endothelial cells support and promote the proliferation of T lymphocytes [25], the ability of EMPs to support T lymphocytes proliferation was assessed. After incubating T lymphocytes with EMPs or miR-155-deficient-EMPs for 3 days, the proliferation of T lymphocytes did not vary significantly (Supplementary Figure 3B–3C). Additionally, miR-155 inhibition in EMPs did not influence apoptosis of T lymphocytes. The percentage of total apoptotic T lymphocytes did not change in response to antagomir-155-EMPs compared with antagomir-NC-EMPs (Supplementary Figure 3A). Consistently, the apoptosis events including BCL-2, BAX and CASPASE-3 expression were not significantly changed (Supplementary Figure 3D). These results suggested that delivery of miR-155 by EMPs did not affect proliferation and apoptosis of recipient T lymphocytes.

### Inhibition of miR-155 in EMPs promoted Th2 and Treg cells but suppressed Th1, Th9 and Th17 cell differentiation

**Figure 3:**
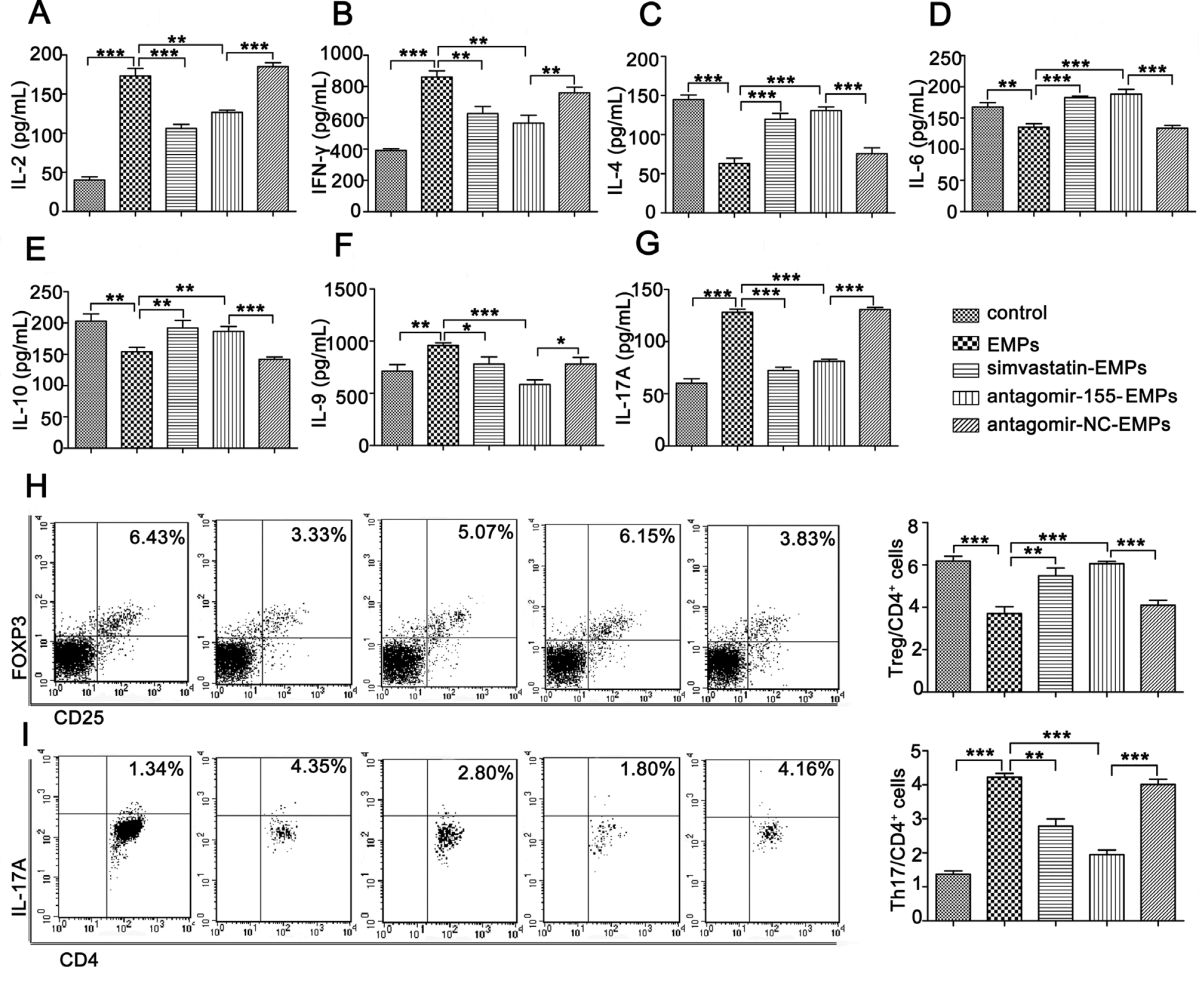
miR-155 in EMPs promotes Th1, Th9 and Th17 and inhibit Th2 and Treg cells differentiation Purified T lymphocytes were co-cultured with 10 μg/mL EMPs from EA.hy926, TNF-α-stimulated EA.hy926, TNF-α-stimulated EA.hy926 protected by 2.5 μmol/L simvastatin, TNF-α-stimulated EA.hy926 transinfected by antagomir-155 and TNF-α-stimulated EA.hy926 transinfected by antagomir-NC, respectively. Cytokines were determined using ELISA kits 3 days after co-culture. T lymphocytes subsets were analyzed by flow cytometry. Data represent mean ± SD from three independent experiments. *P < 0.05, ^*^P < 0.01, ^**^*P < 0.001. (**A**–**B**) Th1-related cytokines including IL-2 (A) and IFN-γ (B). (**C**–**E**) Th2-related cytokines including IL-4 (C), IL-6 (D), IL-10 (**E**–**G**). Th9- and Th17-related cytokines including IL-9 (F), IL-17A (G). (**H**–**I**). (H) CD4^+^CD25^+^FOXP3^+^Treg and (I) CD4^+^IL-17A^+^Th17 cells out of all CD4^+^ cells.

Th1, Th9 and Th17 cells are considered pathogenic and Th2 and Treg cells are considered protective during the course of aGVHD. Therefore, we evaluated whether miR-155 in EMPs modulated T lymphocytes differentiation. As expected, miR-155 inhibition in EMPs substantially suppressed Th1 (IL-2 and IFN-γ), Th9 (IL-9) and Th17-related (IL-17A) cytokines compared to antagomir-NC treatment (Figure 3A–3B, 3F–3G). In contrast, Th2-related cytokines (IL-4, IL-6 and IL-10) were significantly elevated in the supernatants of T lymphocytes treated with miR-155-deficient-EMPs (Figure 3C–3E). We monitored the kinetics of T lymphocytes in response to miR-155-deficient-EMPs and found that frequency of Treg cells was significantly increased (Figure 3H), whereas IL-17A-expressing CD4^+^T cells were markedly decreased (Figure 3I). Therefore, increased Th1, Th9 and Th17 cells and suppression of Th2 and Treg cells might be the primary cause of aGVHD mediated by EMPs-delivered miR-155.

### Abnormal expression of miR-155 in MPs, plasma and T lymphocytes from aGVHD mice

**Figure 4:**
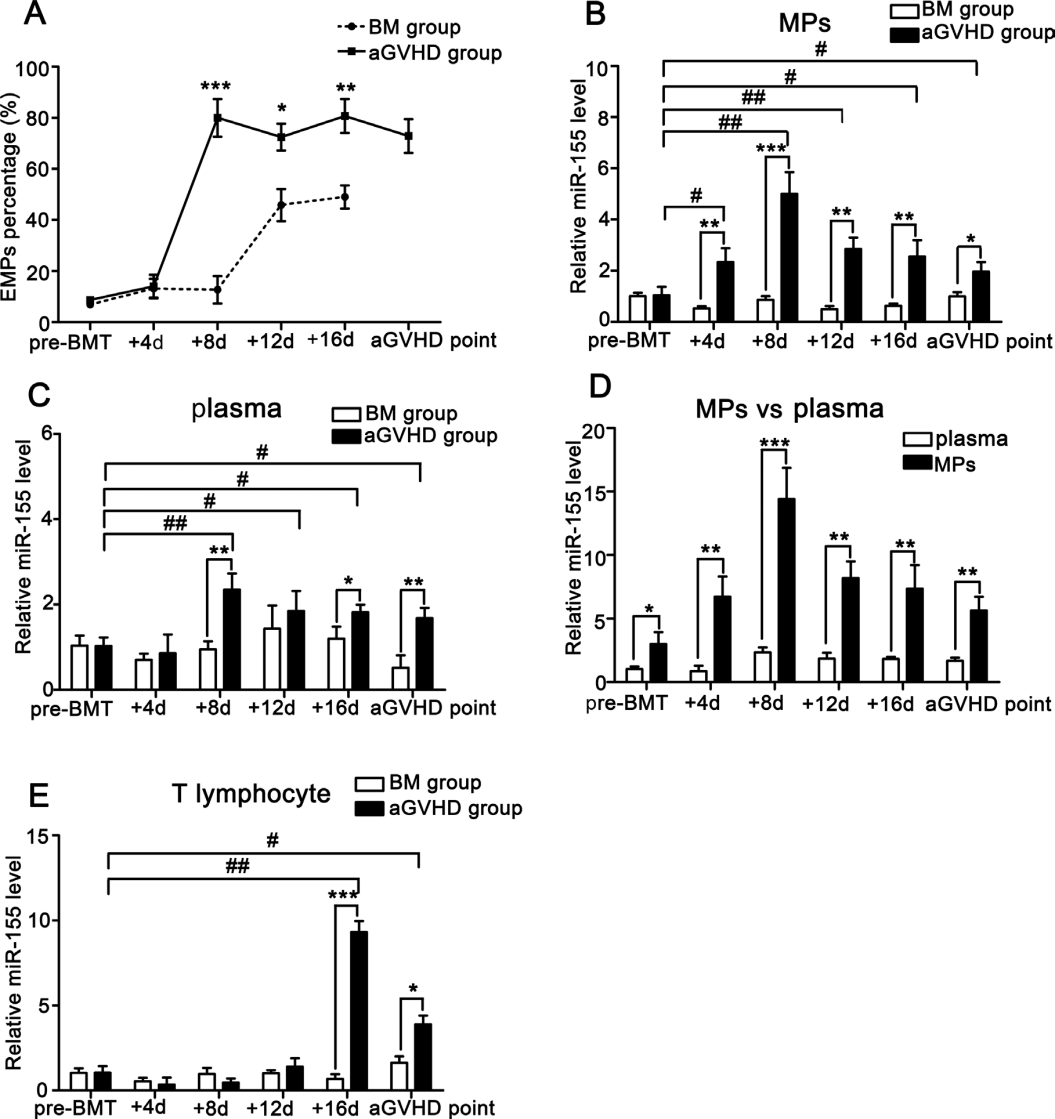
miR-155 expressions in MPs, plasma and T lymphocytes of peripheral blood from BM and aGVHD mice before allo-BMT, at +4d, +8d, +12d, +16d and aGVHD point Average values ± SD from three independent experiments were plotted. (**A**) EMPs isolated from the peripheral blood of BM and aGVHD groups were assessed by flow cytometry analysis. *P < 0.05, ^*^P < 0.01, ^**^*P < 0.001. (**B**) miR-155 levels in MPs isolated from the peripheral blood of BM and aGVHD mice were compared. *P < 0.05, ^*^P < 0.01, ^**^*P < 0.001. Expressions of miR-155 on +4d, +8d, +12d, +16d and aGVHD point were compared with pre-BMT level. ^#^*P <* 0.05, ^##^*P <* 0.01. (**C**) Expressions of miR-155 in plasma from BM and aGVHD mice were compared. *P < 0.05, ^*^P < 0.01. miR-155 levels in plasma at +4d, +8d, +12d, +16d and aGVHD point were compared with pre-BMT level. ^#^*P <* 0.05, ^##^*P <* 0.01. (**D**) miR-155 levels in MPs and plasma at indicated time points were compared in aGVHD mice. *P < 0.05, ^*^P < 0.01, ^**^*P < 0.001. (**E**) miR-155 in T lymphocytes from peripheral blood of BM and aGVHD mice was compared. *P < 0.05, ^**^*P < 0.001. miR-155 in T lymphocytes at +4d, +8d, +12d, +16d and aGVHD point were compared with pre-BMT level. ^#^*P* < 0.05, ^##^*P <* 0.01.

To compare miR-155 levels in MPs, plasma and T lymphocytes isolated from peripheral blood of mice during the course of aGVHD, a well-established MHC-mismatched BMT model was used. We firstly isolated and cultured the primary aortic endothelial cells from BM and aGVHD mice, and then collected the EMPs from cell supernatants and measured the total content of the secreted EMPs by flow cytometry. We found that the amount of EMPs from the peripheral blood of non-aGVHD mice was too low to be detected. However, the amount of EMPs from aGVHD mice was found to be mildly elevated (see P2 in the Supplementary Figure 4). Flow cytometry analysis demonstrated that EMPs from peripheral blood of aGVHD mice were significantly elevated from +8d and slightly decreased at aGVHD point compared with non-aGVHD mice (Figure 4A). Similarly, the miR-155 levels in MPs from aGVHD mice were significantly higher on +8d, +12d, and +16d and slightly lower at aGVHD point compared with control mice (Figure 4B). Furthermore, the miR-155 levels in MPs were remarkably higher than in plasma (Figure 4C–4D). The miR-155 levels in T lymphocytes of aGVHD mice were not higher than those in non-aGVHD mice only until the occurrence of aGVHD (Figure 4E). These data were consistent with the results of clinical aGVHD patients.

### Inhibition of miR-155 reverses the exacerbation of severe murine aGVHD induced by high concentration of EMPs

**Figure 5:**
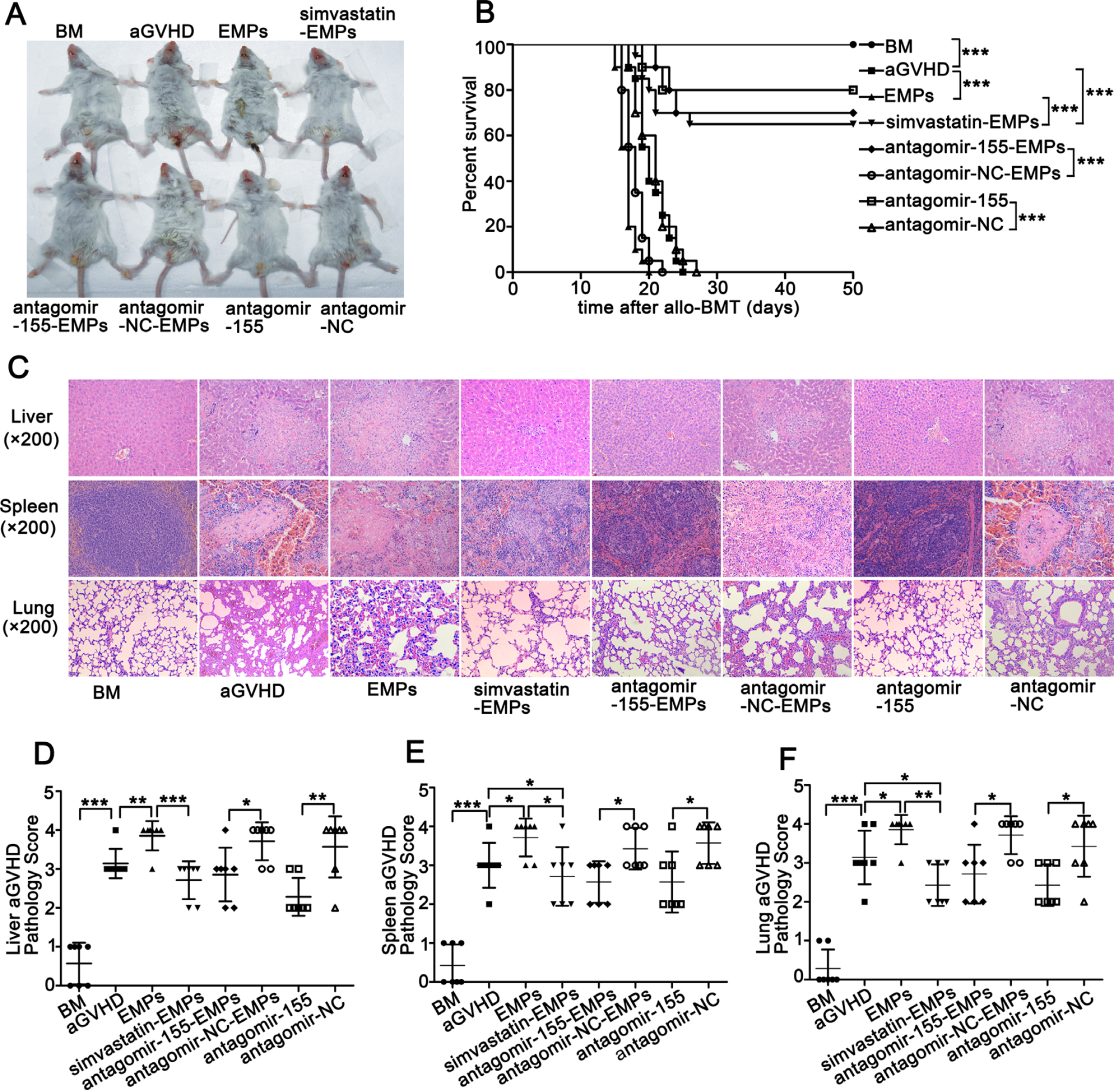
Suppression of miR-155 in EMPs by antagomir-155 ameliorates the exacerbated aGVHD induced by high concentration of EMPs After being subjected to lethal total body irradiation, BALB/c mice were transplanted intravenously with 1 × 10^7^ BM cells and 2 × 10^7^ spleen cells isolated from C57BL/6 donors to establish aGVHD model. EMPs (60 μg) isolated from mouse TNF-α-stimulated MAECs, TNF-α-stimulated MAECs protected by 2.5 μmol/L simvastatin, TNF-α-stimulated MAECs transfected by antagomir-155, TNF-α-stimulated MAECs transfected by antagomir-NC were given intravenously to aGVHD mice on day0 and +7d, retrospectively. Antagomir-155 and antagomir-NC of 25 mg/kg was given intravenously on +7d followed by 5 mg/kg twice weekly up to +21d. (**A**) Representative photograph of clinical severity of aGVHD in each group. (**B**) Survival of recipients was compared using the Mantel-Cox log-rank test. Shown is the mean ± SD from three combined independent experiments. ^**^*P < 0.001. (**C**) Liver, spleen and lung were harvested at +21d and representative histological analysis of hematoxylin-eosin (H&E) staining was shown (original magnification ×200). (**D**–**F**) H&E-stained slides of liver (D), spleen (E) and lung (F) were scored for histopathologic damage and lymphocyte infiltration. Values are means ± SD. *P < 0.05, ^*^P < 0.01, ^**^*P < 0.001.

To examine the contribution of EMPs produced by injured MAECs to the development of aGVHD, we additionally administered high concentration of EMPs to aGVHD mice. Systemic EMPs administration caused more aggressive aGVHD with significantly enhanced mortality and higher clinical score compared with aGVHD group (Figure 5A–5B and Supplementary Figure 5). Histological analysis of the liver, spleen and lung revealed increased aGVHD severity in high concentration of EMPs-treated aGVHD mice (Figure 5C). In contrast, the overall manifestation, the survival and severity of aGVHD assessed by histological grade were significantly improved in aGVHD mice that received miR-155-deficient-EMPs compared with that received antagomir-NC-EMPs and aGVHD group (Figure 5B, 5D–5F). These data demonstrated that inhibition of miR-155 in EMPs significantly attenuated the clinical severity of aGVHD, the histopathology of aGVHD-involved organs and the overall mortality from aGVHD after allo-BMT.

Because previous results suggested that endothelial cell protector simvastatin suppressed EMPs production [26], we evaluated whether simvastatin administration had a salutary effect on EMPs-induced aGVHD aggravation. As expected, mice that received EMPs from simvastatin-protected MAECs exhibited decreased mortality and less severe aGVHD compared with EMPs-treated aGVHD group and aGVHD group (Figure 5A–5B). Furthermore, the severity of histopathological aGVHD was significantly reduced in mice that received EMPs from simvastatin-protected MAECs (Figure 5C–5F). These data demonstrated that prevention of endothelial injury and suppression the production of EMPs significantly improved overall manifestation, prolonged the survival of aGVHD mice and decreased their histopathological severity.

### Inhibition of miR-155 in EMPs significantly influences T lymphocytes differentiation in aGVHD mice

To verify the ability of EMPs to carry miR-155 to recipient T lymphocytes *in vivo*, we respectively studied miR-155 expression in T lymphocytes isolated from peripheral blood of recipient mice. Consistent with *in vitro* results, we found that T lymphocytes in high concentration of EMPs-treated aGVHD mice had remarkably higher miR-155 expression compared to aGVHD group. In contrast, antagomir-155 reversed the abnormal level of miR-155 in T lymphocytes induced by high concentration of EMPs to normal (Supplementary Figure 6). Moreover, we measured the miR-155 levels in CD4+ and CD8+ T cells after they were isolated from peripheral blood of aGVHD mice by magnetic bead separation, and we found that the levels of miR-155 in both CD4+ and CD8+ T cell populations treated with miR-155-deficient-EMPs was remarkably lower than in antagomir-NC-transferred group (Supplementary Figure 7A–7B).

**Figure 6:**
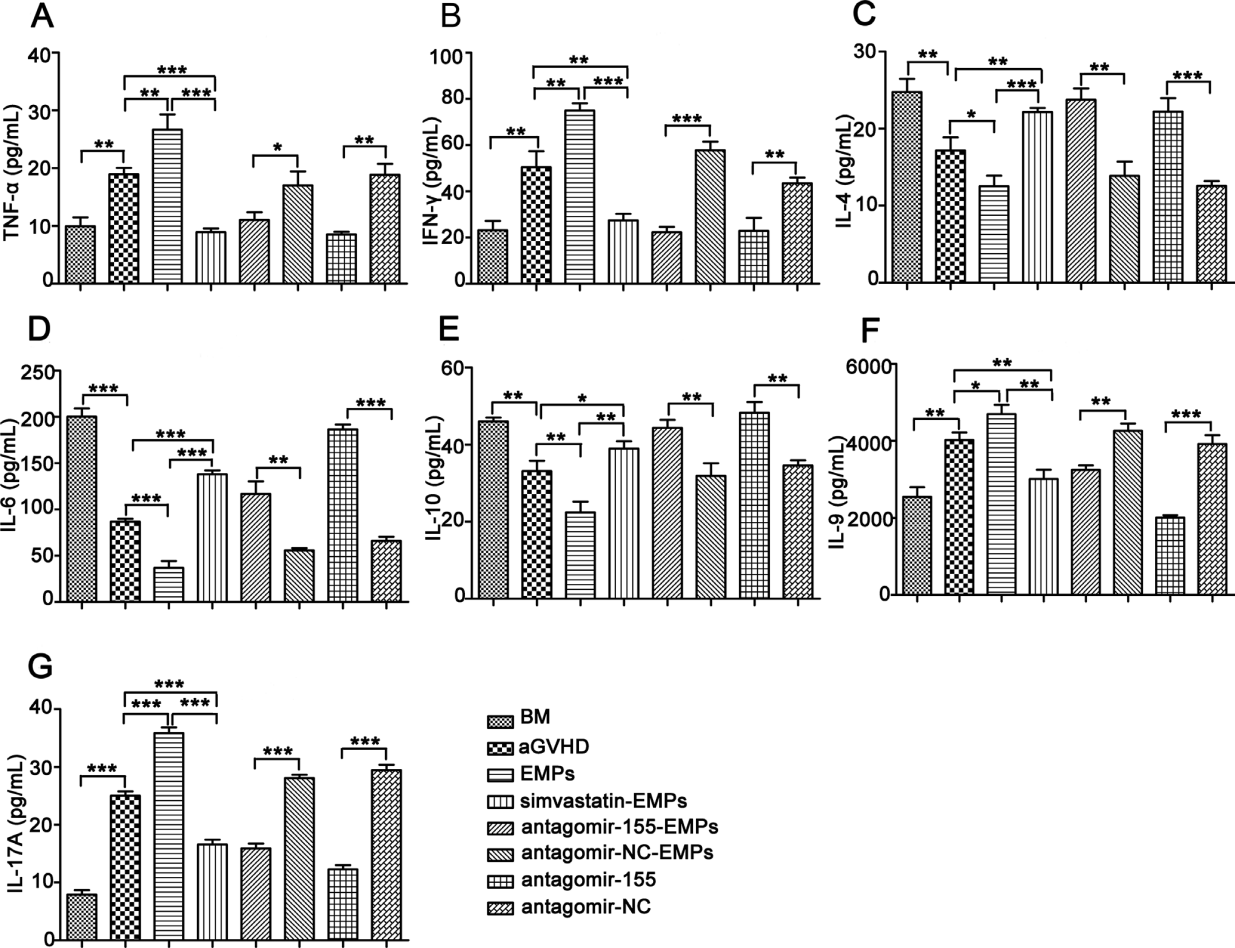
Inhibition of miR-155 in EMPs influences serum cytokine profiles in aGVHD mice After being subjected to lethal total body irradiation, BALB/c mice were transplanted intravenously with 1 × 10^7^ BM cells and 2 × 10^7^ spleen cells isolated from C57BL/6 donors to establish aGVHD model. EMPs (60 μg) from mouse TNF-α-stimulated MAECs, TNF-α-stimulated MAECs protected by 2.5 μmol/L simvastatin, TNF-α-stimulated MAECs transfected by antagomir-155, TNF-α-stimulated MAECs transfected by antagomir-NC were given intravenously to aGVHD mice on day0 and +7d, retrospectively. Antagomir-155 and antagomir-NC of 25 mg/kg was given intravenously on +7d followed by 5 mg/kg twice weekly up to +21d. Serum was taken from recipient mice on +21d and cytokines production were measured by ELISA. Shown is the mean ± SD from three combined independent experiments. *P < 0.05, ^*^P < 0.01, ^**^*P < 0.001. (**A**–**B**) Expressions of Th1-related cytokines including TNF-α (A) and IFN-γ (B). C-E. Th2-related cytokines including IL-4 (**C**), IL-6 (**D**) and IL-10 (**E**–**G**). Th9-, Th17-related cytokines including IL-9 (F) and IL-17A (G).

**Figure 7:**
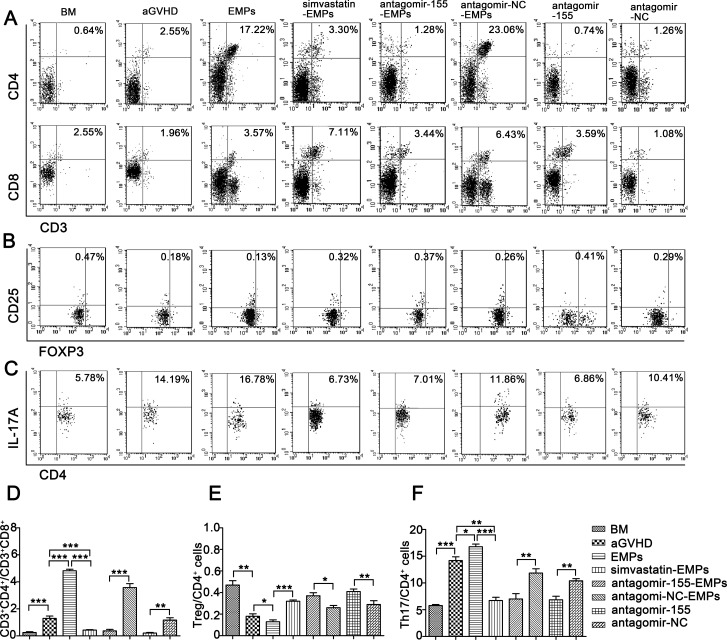
Inhibition of miR-155 in EMPs influences T lymphocytes differentiation in aGVHD mice After being subjected to lethal total body irradiation, BALB/c mice were transplanted intravenously with 1 × 10^7^ BM cells and 2 × 10^7^ spleen cells isolated from C57BL/6 donors to establish aGVHD model. EMPs (60 μg) from mouse TNF-α-stimulated MAECs, TNF-α-stimulated MAECs protected by 2.5 μmol/L simvastatin, TNF-α-stimulated MAECs transfected by antagomir-155, TNF-α-stimulated MAECs transfected by antagomir-NC were given intravenously to aGVHD mice on day0 and +7d, retrospectively. Antagomir-155 and antagomir-NC of 25 mg/kg was given intravenously on +7d followed by 5 mg/kg twice weekly up to +21d. Peripheral blood T lymphocytes subsets were assessed by flow cytometry analysis on +21d. Shown is the mean ± SD from three combined independent experiments. *P < 0.05, ^*^P < 0.01, ^**^*P < 0.001. (**A**, **D**) percentages of CD3^+^CD4^+^ and CD3^+^CD8^+^ cells (**B**, **E**) CD4^+^CD25^+^FOXP3^+^Treg out of all CD4^+^ cells (**C**, **F**) CD4^+^IL-17A^+^Th17 cells out of all CD4^+^ cells.

To investigate the contribution of miR-155 carried by EMPs on the immune function of T lymphocytes in the development of aGVHD, we examined the kinetics of T lymphocytes-related cytokines and subsets in peripheral blood of recipient mice. The characteristic cytokines produced by Th1 (Figure 6A–6B), Th9 (Figure 6F) and Th17 cells (Figure 6G) were elevated in the serum from high concentration of EMPs-treated aGVHD group, whereas a significant reduction was observed in Th2-related cytokines (Figure 6C–6E). As anticipated, antagomir-155 treatment reversed the imbalance in Th1-, Th9-, Th17- and Th2-related cytokines induced by high concentration of EMPs. Flow cytometry analysis showed that the proportion of CD8^+^ T cells was significantly increased, leading to a decreased CD4^+^/CD8^+^ T-cell ratio in miR-155-deficient-EMPs treated mice compared with EMPs-treated aGVHD and aGVHD group (Figure 7A, 7D). Moreover, IL-17A-expressing CD4^+^ T cells were markedly reduced (Figure 7C, 7F) and CD4^+^CD25^+^FOXP3^+^ Treg cells (Figure 7B, 7E) were increased in mice received miR-155-deficient-EMPs. These data indicated that miR-155 delivered by EMPs played a critical role in the initiation of mouse aGVHD by promoting Th1, Th9 and Th17 responses and repressing the expansion of Th2 and Treg cells.

## DISCUSSION

In this study, for the first time our group explored the intricate interactions between endothelial cells and T lymphocytes through the encapsulation of miR-155 by EMPs. Surprisingly, our findings demonstrated that EMPs endogenously released from injured endothelium constituted an effective means of communication between endothelial cells and T lymphocytes and these vesicles were capable of delivering their functional miR-155 into the cytosol of T lymphocytes to promote the development of aGVHD. Specific blockade of miR-155 in EMPs induced defective T lymphocytes differentiation toward Th1, Th9 and Th17 and skewed differentiation toward Th2 and Treg cells, resulting in ameliorated aGVHD manifestation. Taken together, these results suggested that miR-155 carried by EMPs was a superior target for the prevention and treatment of life-threatening aGVHD after allo-HSCT.

EMPs are often-neglected cell membrane vesicles derived from injured peripheral blood endothelial cells during the course of allo-HSCT [27]. Herein, we demonstrated that EMPs were significantly elevated in aGVHD patients and mice, which was consistent with previous results of aGVHD patients [8, 9]. Additionally, we demonstrated that treatment of aGVHD mice with high concentration of EMPs from injured endothelium resulted in exacerbated clinical manifestations of aGVHD that could be reversed by administration of endothelial cell protector simvastatin. Therefore, we speculated that EMPs could be used not only as a diagnostic and prognostic surrogate marker but also as a potential novel therapeutic target for aGVHD.

Zeng et al. have suggested that circulating endothelial cells elevated a little earlier than suppression of the immune system [28]. Our study found that elevated EMPs in the peripheral blood of aGVHD mice and patients preceded the peak in CD4^+^/CD8^+^ T lymphocytes. These findings indicated that EMPs might participate in the initiation of aGVHD by modulating the inflammatory processes of T lymphocytes. Recently, EMPs were found to regulate Th1/Th2 differentiation and functions in patients with acute coronary syndrome [25]. Importantly, our study demonstrated that EMPs could enter into and modulate the functions of T lymphocytes, suggesting that EMPs participated in the pathogenesis of aGVHD via the synergism of T lymphocytes. Although previous studies demonstrated that endothelial cells and EMPs were extremely important for the development of aGVHD [8–10, 29], our study firstly investigated the possible association between endothelial damage and T lymphocytes through EMPs and provided a novel pathomechanism of aGVHD.

The pathogenic role of miR-155 in aGVHD was reported by Ranganathan et al, who demonstrated that miR-155 expression was up-regulated in T lymphocytes from aGVHD mice [19]. However, the initial origin of miR-155 in T lymphocytes was not explored. Notably, our study provided compelling evidences that the increment of miR-155 in EMPs occurred prior to the increase in T lymphocytes and that miR-155 level in T lymphocytes did not increase until the initiation of aGVHD, which indicated that miR-155 in the effector T lymphocytes at the aGVHD attack might originate from EMPs. Meanwhile, we visualized the transport of fluorescently-labeled miR-155 via EMPs shed from TNF-α-stimulated EA.hy926 cells into recipient T lymphocytes. MPs have been proven to transfer microRNAs to selected targeted cells and influence their biological behavior [30, 31]. Fabbri et al identified intercellular microRNA communication through exosomes secreted by tumor cells that carried miR-21 and miR-29b to bind to and activate Toll-like receptors on immune cells, thereby triggering an inflammatory response in the recipient cells and ultimately promoting tumor growth and metastasis [32]. Zernecke et al. showed that the apoptotic bodies shed by endothelial cells enriched in miR-126 were taken up by neighboring vascular cells and subsequently promoted the proliferation of targeted cells [33]. Therefore, our studies indicated that miR-155 delivered by EMPs could serve as an active messenger to regulate the signaling pathways in recipient T lymphocytes to initiate aGVHD. Thus, targeting miR-155 might represent a novel platform to modulate aGVHD after allo-HSCT.

MPs have been proved to protect their selective packaged microRNAs from degradation and are important for potential microRNAs enrichment and transport vesicles in circulation [12, 34]. Pioneering studies indicated that the predominant plasma microRNAs were associated with MPs, while only small amounts of microRNAs were detected in MPs-free plasma. Similar to Xie et al. [35], we found that the miR-155 expression in plasma was significantly elevated in aGVHD patients and was correlated with severity of aGVHD. Moreover, we showed that similar dynamic elevation of miR-155 in MPs was much more obvious than in plasma. Our *in vitro* experiments revealed that miR-155 expression in EMPs was significantly higher than in EA.hy926 cells. Thus, we hypothesized that the microstructure of EMPs played an important role in protecting and concentrating miR-155 to warrant the pivotal role of this inflammatory-related microRNA in the pathological process of aGVHD. Furthermore, our results recommended miR-155 in EMPs as a more sensitive indicator for the diagnosis of aGVHD after allo-HSCT.

miR-155 appears to play a dominant role as a marker for graft rejection. miR-155 has been found highly expressed in T lymphocytes during murine acute cardiac allograft rejection, and miR-155 inhibition *in vitro* suppresses T lymphocytes expansion [36]. Intriguingly, we found that EMPs did not influence proliferation, apoptosis and apoptosis-related proteins via miR-155 delivery, indicating that miR-155 carried by EMPs primarily manipulated the functions of T lymphocytes required for participation in aGVHD. Recently, miR-155 was shown to promote the development of inflammatory T cells including Th1 and Th17 cells [37, 38]. CD4^+^ T cells with high miR-155 levels are prone to develop into Th1 cells, whereas CD4^+^ T cells with low miR-155 develop into Th2 cells that produce anti-inflammatory or immunosuppressive cytokines [39]. Our *in vivo* and *in vitro* experiments simultaneously showed that specific inhibition of miR-155 in EMPs targeted T lymphocytes for defective differentiation toward Th1, Th9 and Th17 and skewed differentiation toward Th2 and Treg phenotypes, which caused ameliorated clinical and pathological manifestations of aGVHD. Regulating or controlling Th1 cytokines could potentially inhibit the development or reduce the severity of aGVHD, whereas increasing Th2 populations plays an important role in the regulation of aGVHD and the mediation of graft-versus-leukemia (GVL) effect. Recent studies showed that polarized Th17 cells induced lethal aGVHD, whereas Treg cells played a pivotal role in protection against the development of fatal aGVHD [40, 41]. Reversing the imbalance of Th17 to Treg cells could prevent aGVHD without offsetting the GVL effect [42, 43]. Therefore, suppression of the delivery of miR-155 by EMPs might be a useful way to control aGVHD while preserving GVL effect. Intriguingly, because miR-155 can act as an oncomir and a pivotal factor in the carcinogenic process in many neoplastic diseases [17, 44, 45], we anticipate that the blockade of miR-155 in EMPs will not only alleviate aGVHD but also significantly benefit the control of underlying hematological diseases after allo-HSCT.

In summary, our results demonstrated that EMPs-delivered miR-155 was involved in the initiation of aGVHD through modulating T lymphocytes functions based on the analyses in aGVHD patients, *in vitro* cell culture and aGVHD mouse model. Our studies provide the rationale to establish the significance of miR-155 in EMPs, which might serve as an early sensitive predictive index and intervention target of aGVHD. However, the signaling pathways by which miR-155 in EMPs modulates the function of T lymphocytes need to be elucidated in future studies.

## MATERIALS AND METHODS

### Patients and sample preparation

We collected all the samples after obtaining approval by the Ethics Committee of Huazhong University of Science and Technology (No: IORG0003571) and obtaining written informed consent in accordance with the Declaration of Helsinki.

Peripheral blood samples were collected at indicated time points (pre-HSCT, +7d, +14d, +21d, +28d and aGVHD point) in a prospective manner from individuals undergoing allo-HSCT with or without aGVHD from May 2014 to June 2015. The assessment of aGVHD was based on commonly accepted criteria [46, 47]. Patient characteristics are detailed in Table 1. MPs were isolated from peripheral blood as previously described [8].

**Table 1:** baseline characteristics of patients with aGVHD and non-aGVHD

Characteristics	aGVHD patients(*n* = 28)	non-aGVHD patients(*n* = 30)
Median age, years (range)	27 (11–52)	28 (13–45)
Gender, *n* (%)		
Male	17 (60.7)	16 (53.3)
Female	11 (39.3)	14 (46.7)
Diagnosis of underlying disease, *n* (%)		
AML	10 (35.7)	14 (46.7)
ALL	7 (25.0)	8 (26.7)
CML	4 (14.3)	3 (10.0)
MDS	4 (14.3)	3 (10.0)
HL	3 (10.7)	2 (6.7)
Interval from diagnosis to HSCT, months (range)	6.5 (2–16)	8 (2.5–15)
Disease status at HSCT, *n* (%)		
CR1	19 (67.9)	22(73.3)
≥ CR2	6 (21.4)	6 (20.0)
NR	3 (10.7)	2 (6.7)
Donor type, *n* (%)		
related	22 (78.6)	20 (66.7)
unrelated	6(21.4)	10 (33.3)
Conditioning regimens, *n* (%)		
IDA-BUCY2	8 (28.6)	12 (40.0)
BUCY2	10 (35.7)	8 (26.7)
IDA-TBI-CY	6 (21.4)	7 (23.3)
TBI-CY	4 (14.3)	3 (10.0)
aGVHD regimens, *n* (%)		
CsA+MTX	18 (64.3)	17 (56.7)
CsA+MTX+MMF	4 (14.3)	3 (10.0)
CsA+MTX+MMF+anti-CD25	6 (21.4)	10 (33.3)

### Cell culture and EMPs isolation

The human umbilical vein endothelial cell line EA.hy926 cells was obtained from American Type Culture Collection (ATCC, Manassas, VA, USA) and primary mouse aortic endothelial cells (MAECs) were purchased from Lifeline Cell Technology (LifeScan, Walkersville, USA). Both EA.hy926 cells and MAECs were stimulated with recombinant human or mouse TNF-α (100 ng/mL, R&D Systems, Minneapolis, USA) for 24 hours to produce EMPs. The cell supernatants were collected and centrifuged at 1000 × g for 10 min and then at 3000 × g for 5 min to remove cells and large debris. The cell-free supernatant was centrifuged at 16000 × g for 60 min at 4°C to pellet EMPs. The obtained EMPs were washed in sterile phosphate-buffered saline (pH 7.4) and pelleted again at 16,000 × g for 60 min. Quantification of the EMPs was performed using the Bradford method [24].

### miR-155 silencing by antagomir treatment

Chemically-modified antagomir-155 was used to inhibit miR-155 expression. The sequences were as follows: antagomir-155: 5′-CCCCUAUCACAAUUAGCA UUAA-3′; antagomir-NC: 5′-AAGGCAAGCUGACCC UGAAGUU-3′. The antagomir-155 and antagomir-NC (Ribobio, Guangzhou, China) were transiently transfected into EA.hy926 cells or MAECs at final concentration of 100nM using the transfection reagent riboFECTTMCP (Ribobio, Guangzhou, China) following the manufacturer's protocols. Efficiency of transfection was validated by quantitative reverse transcription-PCR (qRT-PCR).

### Induction of aGVHD and experiment group

C57BL/6 (H-2K^b^, Thy-1.2) and BALB/c (H-2K^d^, Thy-1.2) mice aged 8–12 weeks were purchased from Beijing HuaFukang Bioscience Company (Beijing, China) and were bred under specific pathogen-free conditions. All procedures were approved by the Institutional Animal Care and Use Committee at Huazhong University of Science and Technology (No:S441).

MHC-mismatched BMT procedures were performed as previously established [48]. BALB/c mice were subjected to lethal total body irradiation with a single-dose of 800 cGy X-rays (Philips MG-324, Hamburg, Germany) on day0. Then, the recipients were transplanted intravenously with 1 × 10^7^ BM cells and 2 × 10^7^ spleen cells isolated from C57BL/6 donors to induce aGVHD. Next, experiments were equally divided into 8 groups (*n* = 15): BM group: only received BM cells; aGVHD group: received BM cells and spleen cells; EMPs group: aGVHD mice treated with EMPs from TNF-α-stimulated MAECs; simvastatin-EMPs group: aGVHD mice treated with EMPs from TNF-α-stimulated MAECs protected by 2.5 μmol/L simvastatin (SelleckChemicals, Houston, USA); antagomir-155-EMPs group: aGVHD mice treated with EMPs from TNF-α-stimulated MAECs previously transfected with antagomir-155; antagomir-NC-EMPs group: aGVHD mice treated with EMPs from TNF-α-stimulated MAECs previously transfected with antagomir-NC; antagomir-155 group: aGVHD mice treated with antagomir-155; antagomir-NC group: aGVHD mice treated with antagomir-NC. Antagomir-155 or antagomir-NC was administered at a loading dose of 25 mg/kg on +7d followed by 5 mg/kg intravenously twice weekly up to +21d post-transplant. High concentration of EMPs (60 μg/d) was isolated from mouse TNF-α-stimulated MAECs and administered to mice intravenously on day0 and +7d. Recipient mice were weighed 4 times a week and monitored daily for clinical signs of aGVHD and survival. The degree of systemic aGVHD was assessed by a scoring system as previously described [49]. Sections of liver, spleen and lung were stained with hematoxylin&eosin and scored using a histopathology scoring system [50].

### Determination of plasma EMPs levels

Mouse peripheral bloods were collected at indicated time points (pre-BMT, +4d, +8d, +12d, +16d and aGVHD point) and centrifuged at 2500 × g for 15 min to prepare plasma. MPs were isolated as previously discribed [8]. Isolated MPs were quantified using FACScan flow cytometer (BD Biosciences, San Diego, USA). 0.8 μm Latex beads (Sigma, Saint Louis, Missouri, USA) were used for the initial gating and as an internal control to measure the MPs before each experiment. PE-labeled CD62E and FITC-labeled CD31 (BD Biosciences, San Diego, USA) were used to identify EMPs.

### Isolation of mouse Aortic Endothelial Cells and quantitative detection of EMPs

The mouse aortic endothelial cells (MAECs) were respectively isolated from BM and aGVHD mice according to the procedures reported by Kobayashi et al [51]. Cells were first grown and cultured at 37°C in a 100 mm culture dish in M199 (Gibco) medium with endothelial cell growth supplement, heparin, and 20% fetal bovine serum (FBS). The EMPs from cell cultural supernatant were collected and detected by flow cytometry as described above. 0.8 μm Latex beads were used for the initial gating and as an internal control to measure EMPs. And a known number of 3 μm Latex beads were added to each tube and run simultaneously with EMPs samples to quantify the numbers of EMPs using FACScan flow cytometer.

### qRT-PCR

Total RNA was extracted from the cells or tissues using the TRIzol reagent (Invitrogen, Carlsbad, CA, USA) and cDNA was generated using the MultiScribe Reverse Transcriptase (Life Technologies, Carlsbad, CA, USA) following the manufacturer's protocols. Quantitative PCR was performed in triplicate using an AB7500 FAST Real-Time PCR System (Applied Biosystems, Foster City, CA, USA). TaqMan primers for mmu-mir-155 (human: assay ID 001806; mouse: assay ID 000479; TaqMan MicroRNA Assay Kit, Life Technologies) were used to monitor miR-155 expression. miR-155 expression was normalized to the reference gene U6 (assay ID 001973; TaqMan MicroRNA Assay Kit, Life Technologies) and relative expression was calculated using the 2^−ΔΔCt^ method.

### T lymphocytes isolation

Human or mouse peripheral blood mononuclear cells (PBMCs) were isolated from human or mouse peripheral blood samples by standard Ficoll-Hypaque density gradient centrifugation methods. T lymphocytes were prepared from human or mouse PBMCs through negative selection using magnetic bead depletion of non-T lymphocytes with the EasySep human or mouse T lymphocytes isolation kit (Stemcell Technologies, USA) following the manufacturer's instructions. The T lymphocytes purity was assessed by flow cytometry analysis and was found to be greater than 95%.

### Confocal Staining

To show the proof-of-principle experiments that EMPs attached and fused with T cells, T cells were incubated with EMPs derived from TNF-α-stimulated EA.hy926 cells either previously labeled with carboxyfluorescein diacetate succinimidyl ester (CFSE) (5 mM) (Molecular Probes, USA) or transinfected with a miR-155 mimic labeled with PE-Cy3 (Ribobio, Guangzhou, China) for 48 hours at 37°C. After being fixed in 4% paraformaldehyde, the cells nuclear were stained with DAPI (Molecular Probes, USA). The fusion of EMPs and T lymphocytes was observed by confocal laser-scanning microscopy (LSM 510 Meta, Zeiss, Gottingen, Germany).

### Proliferation and apoptosis analyses

Cell proliferation of T lymphocytes was determined by flow cytometry analysis using a 488nm argon-ion laser after being treated with CFSE and co-cultured with 10 μg/mL EMPs for 3 days.

The proportion of cells undergoing apoptosis and necrosis was measured using the Annexin V-FITC Apoptosis Detection kit (BD Bioscience, San Diego, USA) following the manufacturer's protocol 3 days after co-cultured with 10 μg/mL EMPs. Fluorescence was measured by FACScan flow cytometer.

### Western blot

After treatments, the cells were homogenized and lysed. The proteins (30–50 μg) were separated on 8–12% sodium dodecyl sulfate-polyacrylamide gels (SDS-PAGE) and transferred to polyvinylidene fluoride (PVDF) membranes (Millipore, Life Science). The antibodies for immunoblotting included rabbit anti-BCL-2, rabbit anti-CASPASE-3, rabbit anti-BAX (Cell Signaling Technology, Danvers, MA, USA) and rabbit anti-GAPDH (Santa Cruz Biotechnology, Santa Cruz, CA, USA). After blocking and incubations of the membranes with primary antibodies and horseradish peroxidase (HRP)-conjugated secondary antibody (Bio-Rad Laboratories), the blots were visualized with chemiluminescence plus reagent (Millipore Corp, Billerica, MA, USA) and the bands were measured using Image Lab Software (Bio-Rad Laboratories, Hercules, CA, USA).

### Cytokine measurement

The concentrations of IL-2, TNF-α, IFN-γ, IL-4, IL-6, IL-10, IL-9 and IL-17A were quantified in the cell supernatants and mouse serum by Enzyme-linked immunosorbent assay (ELISA) kits following the manufacturer's instructions (R&D Systems, Minneapolis, USA).

### T lymphocytes subset analysis

The following flow cytometry antibodies were used to stain the T lymphocytes subsets, including anti-CD4-FITC, anti-CD25-PerCP-Cy5.5, anti-FOXP3-Alexa-Fluor647, and anti-IL-17A-PE for human T cells and anti-CD3-APC, anti-CD4-PE/Cy7, anti-CD8-FITC, anti-CD25-APC, anti-FOXP3-PE, and anti-IL-17A-PE for mouse T cells (BioLegend, San Diego, USA). Standard flow cytometric surface staining was performed. For intracellular FOXP3 and IL-17A staining, the PBMCs were surface-stained, fixed in fixation/permeabilization buffer (BD Biosciences), washed in Perm/Wash (BD Biosciences), and stained with anti-FOXP3 and anti-IL-17A. The stained cells were analyzed using FACScan flow cytometer.

### Statistical analysis

All data are presented as the mean ± standard deviation (SD). GraphPad Prism 5.0 (San Diego, CA, USA) was used to analyze the results and generate the graphs. The Mantel-Cox log-rank test was used to compare recipient survival among groups. The nonparametric unpaired Student’*t* test was used to compare continuous variables between two independent experimental groups and paired Student’*t* test was used when the level of miR-155 after allo-HSCT was compared with pre-HSCT. One-way analysis of variance (ANOVA) was applied to compare continuous variables in experiments with > 2 groups. *P* values < 0.05 were considered significantly different.

## SUPPLEMENTARY MATERIALS FIGURES



## Abbreviations

aGVHD: acute graft-versus-host disease
allo-HSCT: allogeneic hematopoietic stem cell transplantation
MHC: major histocompatibility complex
EMPs: endothelial microparticles
miR-155: microRNA-155
MAECs: mouse aortic endothelial cells
qRT-PCR: quantitative real-time polymerase chain reaction
BMT: bone marrow transplantation
PBMCs: peripheral blood mononuclear cells
CFSE: carboxyfluorescein diacetate succinimidyl ester
SDS-PAGE: sodium dodecyl sulfate-polyacrylamide gels
PVDF: polyvinylidene fluoride
SD: standard deviation
ELISA: enzyme-linked immunosorbent assay
GVL: graft-versus-leukemia
