# Correction: Endothelial microparticles delivering microRNA-155 into T lymphocytes are involved in the initiation of acute graftversus-host disease following allogeneic hematopoietic stem cell transplantation

**DOI:** 10.18632/oncotarget.20579

**Published:** 2017-08-29

**Authors:** Ran Zhang, Xiaoxiao Wang, Mei Hong, Ting Luo, Miaomiao Zhao, Haorui Shen, Jun Fang, Xiaojie Li, Sibin Zang, Ping Chen, Dimin Nie, Peng Zheng, Qiuling Wu, Linghui Xia

**Affiliations:** ^1^ Institute of Hematology, Union Hospital, Tongji Medical College, Huazhong University of Science and Technology, Wuhan 430022, China

**This article has been corrected:** the institution information was updated to include the postal code.

The authors sincerely apologize for this oversight.

Original article: Oncotarget. 2017; 8:23360-23375. https://doi.org/10.18632/oncotarget.15579

