# Supplementary material for: Endothelial microparticles delivering microRNA-155 into T lymphocytes are involved in the initiation of acute graft-versus-host disease following allogeneic hematopoietic stem cell transplantation
Source: Oncotarget. 2017 Feb 21;8(14):23360–75. doi: 10.18632/oncotarget.15579 (PMC5410310; doi:10.18632/oncotarget.15579)
Supplement: Supplementary file 1 [file oncotarget-08-23360-s001.pdf]

Endothelial microparticles delivering microRNA-155 into T lymphocytes are involved in the initiation of acute graft-versus-host disease following allogeneic hematopoietic stem cell transplantation

Supplementary Materials

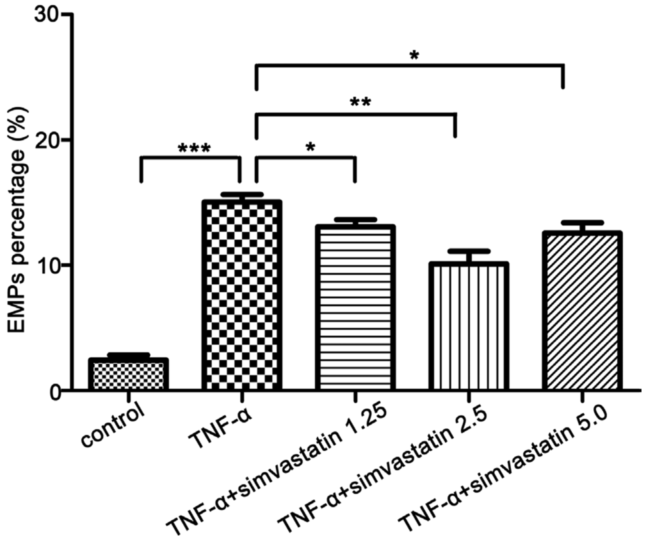

Supplementary Figure 1: EMPs from TNF-α-stimulated EA.hy926 and non-stimulated EA.hy926 cells were quantified by flow cytometry analysis.

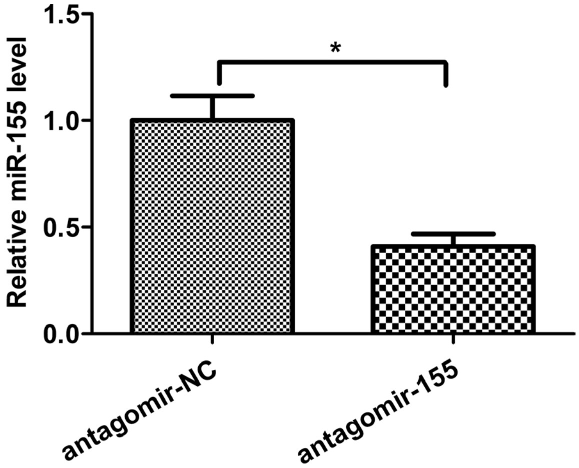

Supplementary Figure 2: Inhibition of miR-155 in endothelial cells influences the miR-155 levels in EMPs.

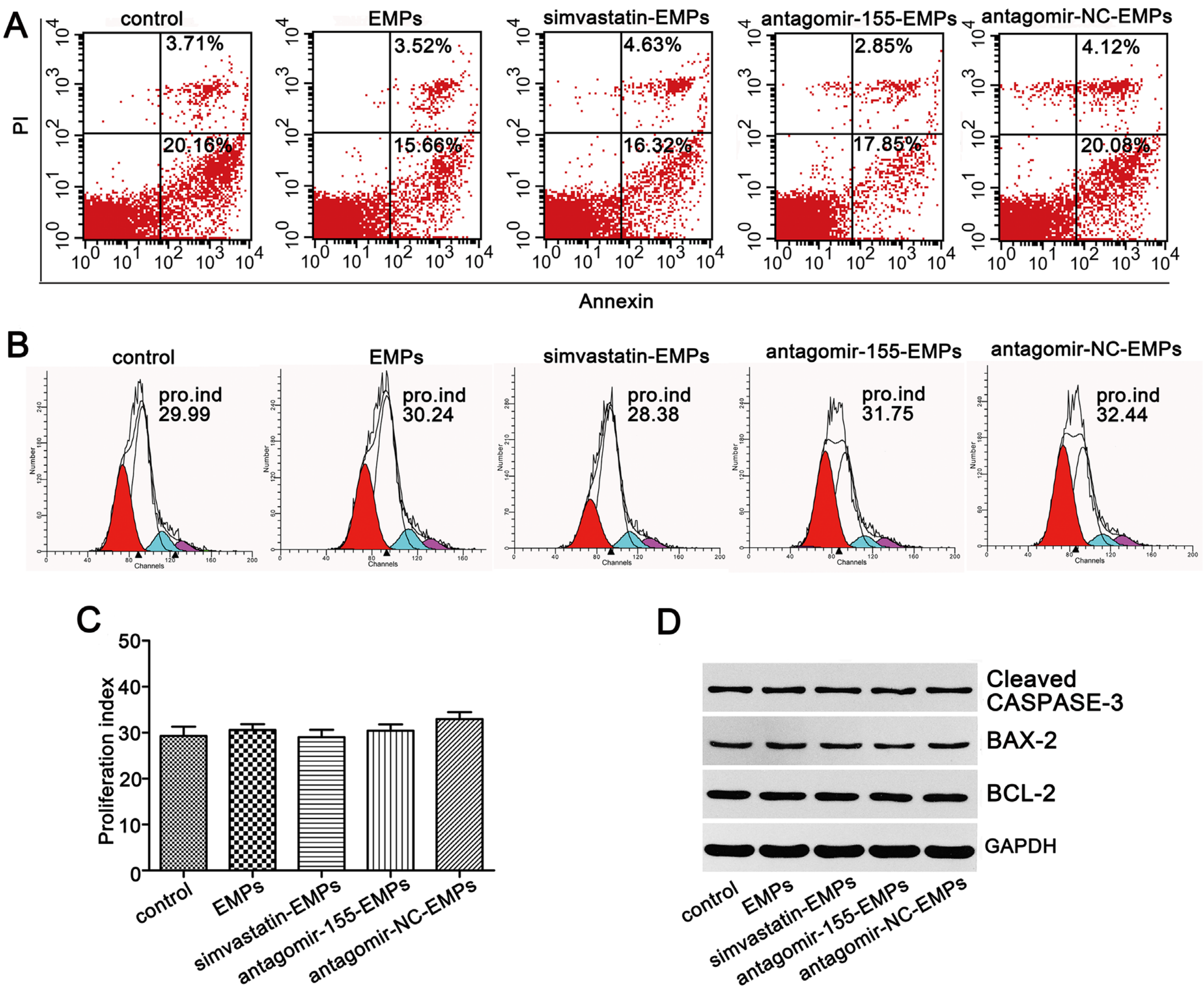

Supplementary Figure 3: miR-155 delivered by EMPs did not influence T lymphocytes proliferation and apoptosis.

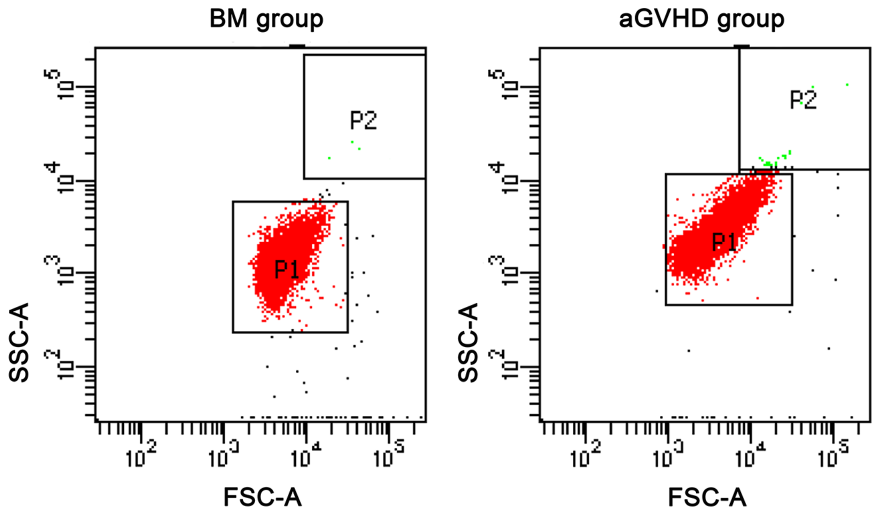

Supplementary Figure 4: Quantitative determination of EMPs from aortic endothelial cells isolated from BM and aGVHD mice.

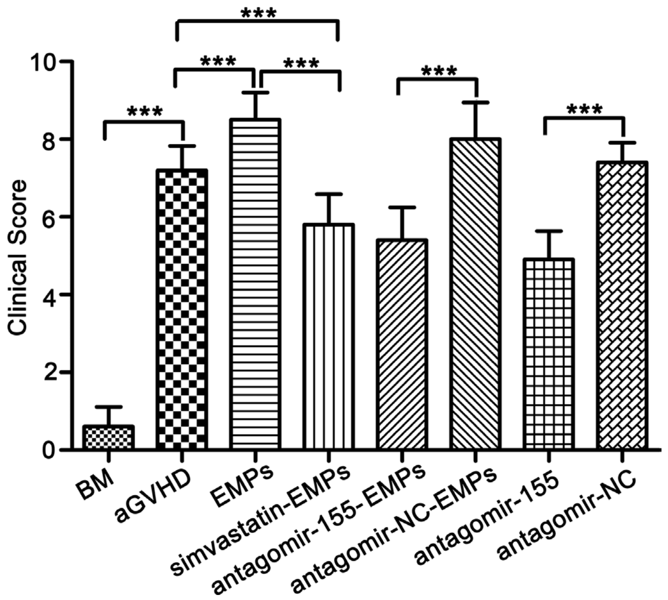

Supplementary Figure 5: Suppression of miR-155 in EMPs improves aGVHD clinical scores induced by high concentration of EMPs.

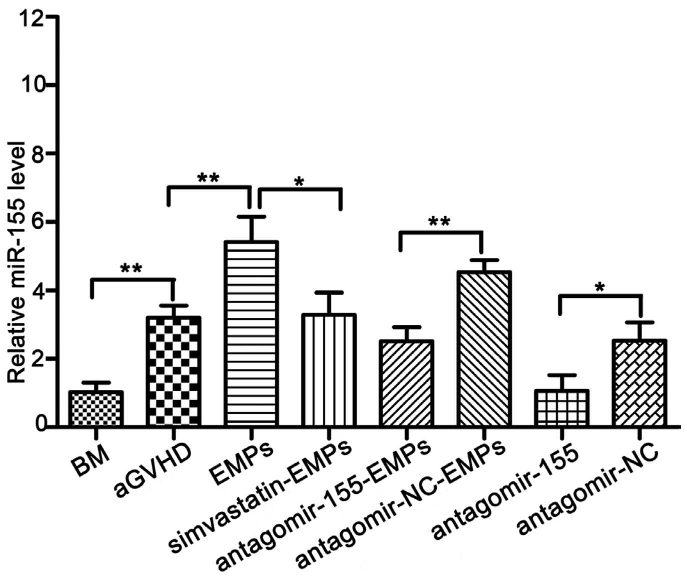

Supplementary Figure 6: Inhibition of miR-155 in EMPs influences the miR-155 levels in T lymphocytes from peripheral blood in aGVHD mice.

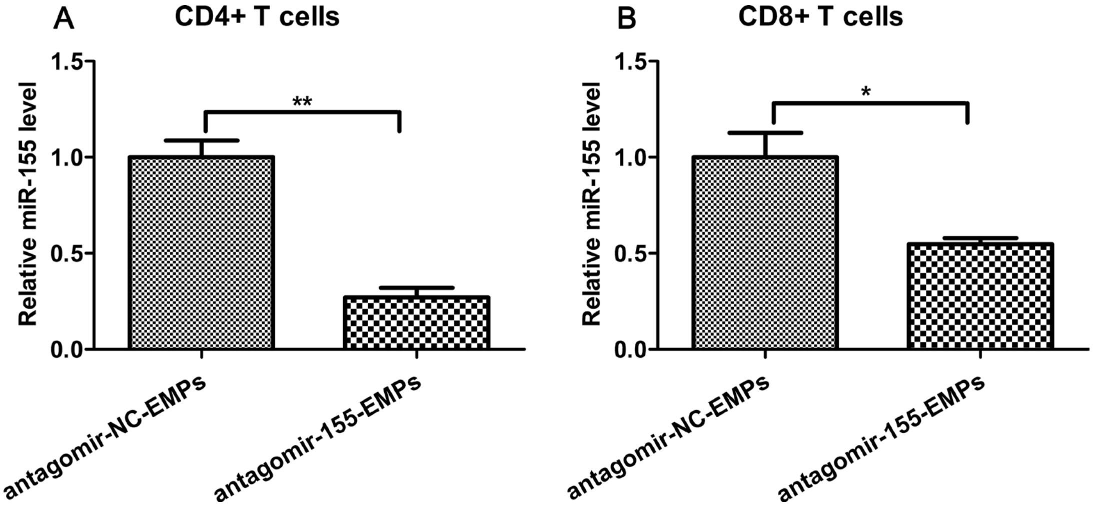

Supplementary Figure 7: Inhibition of miR-155 in EMPs influences the miR-155 levels in CD4+ and CD8+ T lymphocytes from peripheral blood in aGVHD mice.
